# Intellectual Disability in a Birth Cohort: Prevalence, Etiology, and Determinants at the Age of 4 Years

**DOI:** 10.1159/000448912

**Published:** 2016-09-06

**Authors:** Simone M. Karam, Aluísio J.D. Barros, Alícia Matijasevich, Iná S. dos Santos, Luciana Anselmi, Fernando Barros, Sandra Leistner-Segal, Têmis M. Félix, Mariluce Riegel, Sharbel W. Maluf, Roberto Giugliani, Maureen M. Black

**Affiliations:** ^a^Programa de Pós-Graduação em Saúde da Criança e do Adolescente, UFRGS, Porto Alegre, Brazil; ^b^Faculdade de Medicina, Universidade Federal do Rio Grande (FURG), Rio Grande, Brazil; ^c^Programa de Pós-Graduação em Epidemiologia, UFPel, Pelotas, Brazil; ^d^Departamento de Medicina Preventiva, Faculdade de Medicina, Universidade de São Paulo (USP), São Paulo, Brazil; ^e^Serviço de Genética Médica/HCPA, Brazil; ^f^Programa de Pós-Graduação em Genética e Biologia Molecular, UFRGS, Porto Alegre, Brazil; ^g^Programa de Pós-Graduação em Farmácia, UFSC, Florianópolis, Brazil; ^h^Departamento de Genética, UFRGS, Porto Alegre, Brazil; ^i^Department of Pediatrics, University of Maryland School of Medicine, Baltimore, Md., USA; ^j^Department of Epidemiology and Public Health, University of Maryland School of Medicine, Baltimore, Md., USA

**Keywords:** Cohort studies, Developmental delay, Intellectual disability, Mental retardation, Prevalence

## Abstract

**Background:**

Intellectual disability (ID), characterized by impairments in intellectual function and adaptive behavior, affects 1-3% of the population. Many studies investigated its etiology, but few are cohort studies in middle-income countries.

**Aims:**

To estimate prevalence, etiology, and factors related to ID among children prospectively followed since birth in a Southern Brazilian city (Pelotas).

**Methods:**

In 2004, maternity hospitals were visited daily and births were identified. Live-born infants (n = 4,231) whose family lived in the urban area have been followed for several years. At the age of 2 and 4 years, performances in development and intelligence tests were evaluated using the Battelle Developmental Inventory and Wechsler Intelligence Scale, respectively. Children considered as having developmental delay were invited to attend a genetic evaluation.

**Results:**

At 4 years of age, the prevalence of ID was 4.5%, and the etiology was classified into 5 groups: environmental (44.4%), genetic (20.5%), idiopathic (12.6%), neonatal sequelae (13.2%), other diseases (9.3%). Most children presented impairment in two or more areas of adaptive behavior. There was no difference in prenatal care attendance or maternal schooling among the groups.

**Conclusion:**

For about 40% of children, ID was attributed to nonbiological factors, suggesting that the rate may be reduced with appropriate interventions early in life.

## Introduction

Intellectual disability (ID, formerly referred to as ‘mental retardation’) is characterized by a significant impairment in intellectual function, associated with impairment in adaptive behavior: communication, domestic personal care, social skills, use of community resources, autonomy, health and safety, school, leisure, as well as work [1]. The onset of ID occurs before 18 years of age, which characterizes it as a developmental disorder and not as a cognitive disorder such as dementia [1].

The etiologic determination of ID is important for treatment, genetic counseling, family planning and support, as well as surveillance and implementation of preventive programs. It has been estimated that ID prevalence ranges from 1 to 3% [2,3], but wider variation has been reported [3]. The main risk factors for developmental disorders can be divided into direct factors like male gender, maternal age above 30 years, multiple pregnancies, high birth order, low birth weight, small head circumference, preterm birth, iron and iodine deficiencies, brain injury, and malnutrition, as well as indirect factors, like low parental schooling, maternal depression, low socioeconomic status, and inadequate stimulation [4]. The direct and indirect ID costs are estimated to be USD 52 billion a year in the USA [5] and EUR 43.3 billion a year in Europe [6].

Most of the evidence on ID prevalence and etiology comes from high-income countries [7,8]. Furthermore, etiologic investigation is commonly carried out in selected samples, such as clinics or special education schools. In this study, nested in a population-based birth cohort, epidemiologic and clinical data were analyzed which allowed the assessment of ID prevalence and its etiology. Few birth cohort studies have evaluated the prevalence and etiology of ID. Even in high-income countries, etiologic investigation has been usually conducted in selected populations [9]. This is, to our knowledge, the first study in Latin America that assesses the prevalence and etiology of ID using standard criteria and applying clinical as well as laboratory tools. Furthermore, as pointed by Maulik et al. [9], longitudinal data and genetic evaluation of ID cases, mainly from low- and middle-income countries (LMICs), are necessary. The present study was aimed at assessing the prevalence rate and etiology of ID among children who have been prospectively followed since birth in a Southern Brazilian city.

## Materials and Methods

In 2004, maternity hospitals in Pelotas, Brazil, were visited daily, and the births were identified. Live-born infants whose family lived in the urban area of the city were examined and their mothers interviewed (n = 4,231) [10]. Face-to-face interviews were conducted within 24 h after delivery using a standardized precoded questionnaire, which included the following nine sections: identification; delivery and child health; antenatal care and gestational morbidity; reproductive history; maternal characteristics and lifestyle, including amount and frequency of alcohol intake and mean number of smoked cigarettes in each pregnancy trimester; paternal work and family income, as well as laboratory testing during pregnancy [10]. The research team assessed newborn length, cephalic and abdominal circumference, as well as gestational age. The hospital staff measured the birth weight using pediatric scales calibrated with a precision of 10 g, and which were regulated weekly by research staff using standard weights. Maternal medical records (prenatal card) were reviewed for information regarding prenatal care attendance, maternal diseases, use of medications, and previous pregnancies. Nonhospital deliveries (n = 20) were included, as most of these mothers were evaluated at a maternity after delivery [10].

Cohort members were followed up at 3, 12, 24, and 48 months. The 3-month assessment was partially conducted in a research clinic, and other assessments were carried out at participants' home. In each visit, the children were weighed and examined, and their mothers were interviewed. Maternal depressive symptoms were evaluated using the Edinburgh Postnatal Depression Scale (EPDS) [11], which was validated for a Brazilian population [12].

### Developmental and Stimulation Evaluation

Stimulation was assessed at 12, 24, and 48 months using a 5-item questionnaire that evaluated cognitive stimulation markers, parent-child interactions, and interpersonal interactions [13,14]. The questionnaire evaluated whether the child: (1) had someone read or told a story to her/him; (2) went to a park or a playground; (3) went to someone else's house; (4) watched TV, and (5) whether the child had a storybook. Positive answers were summed, yielding a score ranging from 0 to 5.

At 12 and 24 months, child development was assessed using the screening version of the Battelle Developmental Inventory (BDI), which included five domains: personal - social; adaptive; fine and gross motor; communication, and cognitive [15]. In the absence of an adapted version in Portuguese, relevant BDI questions were translated into Portuguese from the Spanish version, pretested, and revised by the investigators for fidelity to the original meaning. Interviewers, who had at least 11 years of schooling, were trained by a pediatrician specialized in child development and were retrained every 2 months to maintain a high level of standardization [13]. Training included becoming familiar with the tests and related procedures, understanding what aspects were under study in each test, and performing the assessment in groups with children in the same age range, until all interviewers achieved the desired skill, and the group as a whole had a standard approach to each test. At 48 months, an abbreviated version of the Wechsler Preschool and Primary Scale of Intelligence (WPPSI), including Arithmetic, Block Design-Picture, Pictures Completion, and Similarities subtests, was administered by trained psychologists [16]. The Argentinean version of the WPPSI was used because the test had not been validated in Brazil. The selection of subtests was based on the high correlation of subtests between the WPPSI and WISC-III scale [17]. Children who were ill on the day of testing were rescheduled to another day. Children with sensory impairments (totally deaf and blind) and with cerebral palsy (CP) were excluded from the WPPSI (4th follow-up). Here, CP is defined according to the classification based on the International Workshop on Definition and Classification of Cerebral Palsy, 2004. Children with CP who had been previously evaluated were not included in the present study.

### Genetic Evaluation

The genetic evaluation was conducted at the age of 7-8 years among children who met at least two of the following criteria:

- BDI <-1 standard deviation (SD) at the 24-month evaluation.
- One or more of the following problems noted by the interviewer at the 48-month visit: communication impairment (comprehension/language); aggressive behavior; no interaction with interviewer, and inability or refusal to complete WPPSI.
- Intelligence quotient (IQ) below 70 points at the 48-month visit, based on the WPPSI scale.
- Fifteen children who did not complete the WPPSI evaluation were also invited to attend the clinical evaluation.

**Fig. 1:**
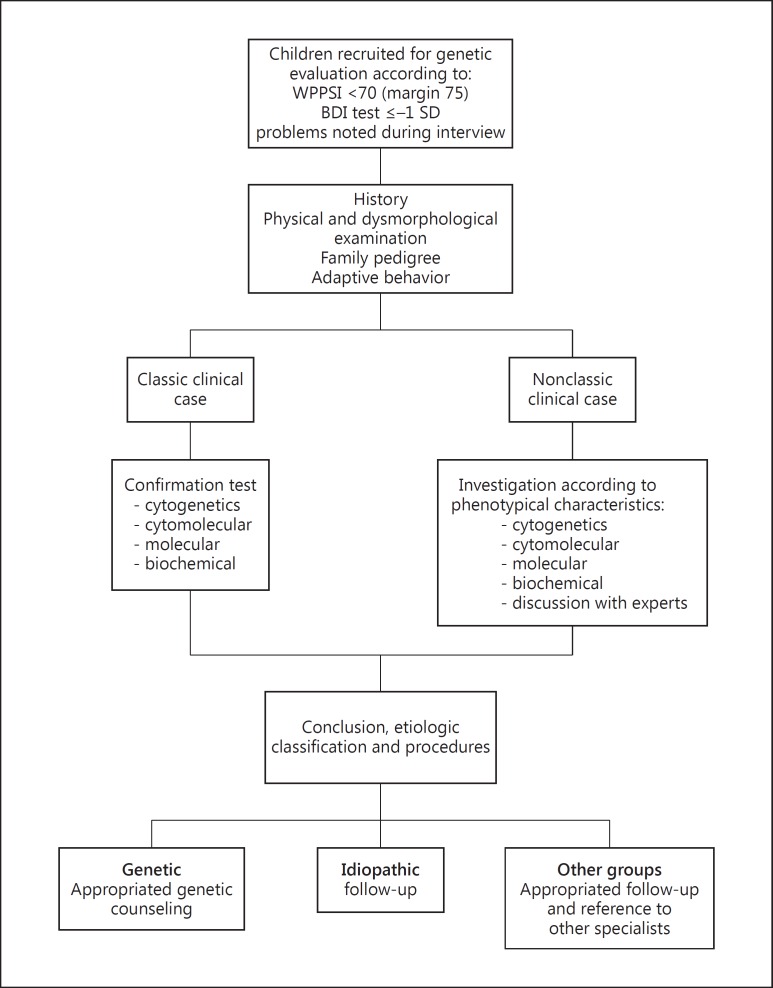
Recruitment for genetic evaluation in the 2004 Pelotas Birth Cohort Study and clinical and laboratory investigations.

Multiple criteria were used to increase screening sensitivity and identify children at risk of ID. Some children met multiple criteria (fig. 1).

Among 195 children who attended the clinic visit, 44 children were excluded from the analysis because their score on the BDI was <-2 SD at 24 months of age. However, at the age of 7-8 years, these same children showed a WPPSI score >70, did not present dysmorphic features or behavioral problems, and their school performance was adequate. These children were considered as having temporary delay, probably related to low birth weight (11.4%), prematurity (25.6%), HIV (13.6%), and 5-min Apgar score <6 (5.7%).

### Clinical Genetic Evaluation

Children with suspected ID were evaluated in a child friendly environment by a pediatrician/clinical geneticist, who collected information on social and demographic family characteristics, child clinical history, and family pedigree, including at least the last three generations. During the mother's interview, the child's interaction with the family, the investigator, and the environment was observed. Furthermore, the clinical geneticist carried out a physical and a dysmorphological examination and evaluated adaptive behavior by asking questions based on the Vineland-II scale [18]. Information on school performance at the age of 7-8 years was also gathered. At the end of the evaluation, a diagnostic hypothesis was made and blood or urine samples were collected, when necessary. The following laboratory tests were requested, according to the diagnostic hypothesis (fig. 1):

- Cytogenetic analysis at 400-500 G-band resolution (GTG karyotype). This analysis is for children who presented a possible ID associated to minor or major anomalies or a classic phenotype syndrome such as trisomy 21. A total of 47 tests were analyzed.
- Molecular cytogenetic tests. These tests, e.g. fluorescent in situ hybridization, were requested when classical microdeletion syndromes were suspected. Five tests were performed.
- Array-based comparative genomic hybridization (array-CGH or a-CGH). This is a whole-genome analysis using the Agilent 60-mer oligonucleotide-based microarrays with a resolution of 60 Kb. It was performed for those subjects whose GTG karyotype was normal, but with a suspicion of structural abnormalities or microdeletion/microduplication syndromes. A total of 31 tests were performed.
- Polymerase chain reaction. This test amplified the region containing a CGG (FRAXA allele) or GCC (FRAXE allele) repeat in fragile X syndrome. Polymerase chain reaction was requested for males (n = 18) with the following characteristics: prominent ears, elongated face, poor visual contact, hypersensibility, macroorchidism, or for males with unexplained ID and normal GTG karyotype, considering the literature recommendations and the fact that the above features could not be present at the age of 7-8 years [2,3].
- Biochemical tests. The following tests were requested for children with suspected metabolic disorders: screening tests for inborn errors of metabolism in urine; gas chromatography for organic acids in a random morning sample; high-performance liquid chromatography of amino acids in plasma and/or urine; serum transferrin in gel isoelectric focusing, and acylcarnitine profiles (liquid chromatography associated with tandem mass spectrometry) in dried blood spots. Biochemical tests (n = 6) were performed based on findings in history and/or clinical examination as recommended [2,3]. Organic acids were requested for children presenting failure to thrive, vomiting episodes, recurrent infections, and previous hypotonia. Transferrin electrophoresis was requested for children with inverted nipples, seizures, autistic behavior, and for whom other causes of ID were excluded.

Children with suspected ID were classified into one of the following groups, according to the possible etiology:

- Genetic: based on clinical and/or laboratory diagnosis of a genetic disorder.
- Idiopathic: those children with syndromal characteristics, such as dysmorphisms and abnormal behavior with normal laboratory tests and inconclusive clinical evaluation.
- Potential neonatal sequelae: history of anoxia based on information gathered from one of the following sources: neonatal card; perinatal questionnaire; hospital records and/or image exams; hypoglycemia; intracranial bleeding; neonatal meningitis; birth injury, or extended hospitalization during the neonatal period associated or not to prematurity.
- Other diseases: children who did not present any complication during delivery or in the first days of life, but were diagnosed as having epilepsy, and visual or hearing impairment.
- Nonbiological causes: no evidence of genetic disorders, neonatal sequelae, syndromal features, or any other disease, combined with socioaffective deprivation and a poor school performance.

Children's relatives (mothers, fathers, or siblings) with suspected ID were also evaluated when familial history suggested that they could be potentially affected. Congenital infections were discarded based on maternal records and children examination.

The institution's Ethics Committee approved this study, and the guardians of all participants signed an informed consent form.

### Statistical Analysis

Analysis was performed using Stata 13.0. The average IQ at 48 months was compared using analysis of variance (ANOVA), the assumption of homogeneity of variances was assessed using Levene's test. χ^2^ test was used to compare proportions. Multinomial logistic regression was used to estimate the risk of being in a category of ID. In this analysis, the nonbiological group was used as a reference.

Concerning the socioeconomic variables, parental schooling was assessed through the total of completed years of formal education, and socioeconomic level was evaluated using the Brazilian Association of Market Research Institutes (ABEP) classification, in which the highest level is designated A and the lowest is E. This classification assesses the possession of household goods and the head's of the household schooling time.

To estimate ID prevalence, the number of children who fulfilled the inclusion criteria (n = 170) was divided by the number of children interviewed at the age of 48 months (n = 3,799), excluding those 44 children with temporary delay.

## Results

For the present study, 214 children with suspected ID were invited to visit the research clinic to be evaluated by a pediatrician/clinical geneticist (S.M.K.). A total of 195 children (91.1%) were evaluated, with an ID prevalence of 4.5%. IQ was assessed in 3,784 children in the total of the cohort study, and the average IQ was 96.6. With respect to ID etiology, among the 195 children who attended clinical evaluation, 44 were excluded because they were considered as having a temporary developmental delay, as previously described. Information on ID etiology was obtained for 151 children. These children were classified in five groups according to ID etiology: genetic, idiopathic, potential neonatal sequelae, other diseases, and nonbiological. Most pregnancies were planned, access to prenatal care was nearly universal, more than 58% of mothers had their first antenatal care visit in the first trimester of pregnancy, and most mothers had seven or more antenatal care visits. Considering the number of previous miscarriages, the difference among groups was not statistically significant (p = 0.14).

Concerning distribution of neonatal characteristics among the five groups (data not shown), birth weight, Apgar score, and gestational age were lower among children with potential neonatal sequelae (p < 0.001).

Table 1 shows that a genetic disorder was responsible for 20.5% of ID cases. The genetic group is characterized by chromosomal abnormalities (n = 12), including Down (n = 7) and Williams syndromes, and four children presented abnormal microarrays; Mendelian inheritance (n = 7), including fragile X and Cornelia de Lange syndromes, tuberous sclerosis, and one family with autosomal dominant microcephaly; and multifactorial inheritance (n = 12), including several congenital defects and attention deficit hyperactivity disorder. More details about this genetic group can be found in another publication [19]. Children in the idiopathic group (12.6%) presented suspected ID with distinguishing characteristics, such as impairments on adaptive behavior, overweight (BMI ≥17.53 in females and 17.71 in males), or dysmorphic features. Potential neonatal sequelae due to hypoglycemia, meningitis, anoxia, and other injuries were considered as an ID cause in 13.2% of the cases. Other conditions, such as sensorineural deafness, epilepsy, as well as visual deficiency were observed in 9.3% of the cases, and 44.4% of the children did not have a biological cause, which explained ID. Children in the potential neonatal sequelae group and those whose ID suspicion was due to genetic disorder showed lower IQ. All children belonging to biological groups of ID (genetic, idiopathic, sequelae, and others diseases) presented impairment in two or more areas of adaptive behavior, such as communication, social skills, and daily living, compared to 13.4% among those in the nonbiological group. The proportion of children attending special education schools was higher among those with genetic disorders (table 1). Most children in the environmental group who were attending general education schools were unable to read and write, and more than half of them presented language problems observed during clinical evaluation or reported by their mothers (data not shown).

Table 2 shows maternal depression, which ranges from 22 to 50%, maternal smoking prevalence, and alcohol intake during pregnancy. Socioeconomic variables presented a similar distribution among the five groups. Multinomial logistic regression shows that the risk of ID was 7.00 times higher among those mothers >35 years old, in comparison to those whose ID was attributable to social causes. Maternal depression from birth to the age of 4 years was associated with a higher risk of having a child on the second category of ID, which includes children with an idiopathic diagnosis, neonatal sequelae, and other diseases (see online suppl. table; for all online suppl. material see www.karger.com/doi/10.1159/000448912).

## Discussion

This study evaluated the prevalence of ID and its etiology in a population that has been prospectively followed since birth. The studied population included almost all live-born infants (99.2%) delivered in 2004, whose family lived in the urban area of Pelotas, and an ID prevalence of 4.5% was found.

Most studies on ID prevalence have been carried out in high-income countries, based on clinic or administrative data, and did not evaluate the etiology of ID [9]. Similarly to our research, a birth cohort study conducted in the UK evaluated ID presence at 7 years of age and found a prevalence of 2.4% [20]. However, other population-based studies have reported a higher prevalence [21,22,23].

Genetic disorders were found in approximately 20% of all investigated children, which is the second most frequent cause of ID, slightly below from what was mentioned in other studies [24]. Down syndrome was a common genetic diagnosis as seen in other cohorts [22,23], and other chromosomal abnormalities and pathogenic copy number variation have been identified as a cause of ID, as found in other studies, which is indicating the need for performing cytogenetic studies, especially chromosomal microarrays [2,3]. The studied children were largely investigated but some experiments, such as exome sequencing, were not available at the time of the research, and they will be analyzed in future follow-up in this cohort. Furthermore, some syndromes, such as fragile X, become more evident as the time passes. It is important to consider that the percentage of genetic ID could have been underestimated, given that some of the idiopathic cases could be due to unidentified genetic disorders. However, for up to 60% of ID there is no identifiable cause [24]. Considering this, the proportion of idiopathic cases is similar to what was previously reported [24]. Potential neonatal sequelae corresponds to about 13% of ID, which is less frequent than in LMICs cohorts, and perhaps it could be attributable to a high antenatal care attendance, including seven or more visits. Different from those studies in LMICs [22,23], even considering genetic causes, consanguinity is not an important risk factor for ID in this population, since there were no consanguineous marriages in our cohort.

More than 40% of children with suspected ID did not have any medical condition that could explain a delayed performance in the intelligence test. Several studies have reported that family income and low maternal schooling are the strongest predictors of developmental delay [25]. Parental socioeconomic status (SES) can affect an individual from uterus throughout life. Stress, nutrition, parental care, and cognitive stimulation are some of the factors that mediate the impact of SES on brain structure and cognitive function, as memory, executive function, and language [26,27,28]. In order to deeply analyze these aspects we used the multinomial logistic regression, which did not find any association of SES or parental education with ID groups. On the other hand, most of the children affected by ID are from low-income families whose parents have a low educational level. This might be due to the effect of poverty on child development and educational outcomes [4,25,26]. Concerning maternal depression, it is difficult to conclude if this finding suggests a cause or a consequence.

This study has some methodological limitations. Data collected in the first 4 years of life were used to identify children with ID at the age of 7-8 years, and it is possible that additional ID cases may have emerged between the ages of 4 and 6-7. Array-CGH and exome sequencing were unavailable for all children and both tests could explain some other cases. Moreover, 8.8% of children with suspected ID were not able to be followed, but these children were equally distributed according to birth weight, gestational age, Apgar score, maternal age, and maternal schooling (data not shown).

Besides the limitations discussed above, this study presents some advantages as the unlikeliness of the selection bias due to a high proportion of successfully followed cohort members [10]. The likelihood of information bias was minimized by collecting data on the major variables, at birth and at early childhood, with a short recalled time. Multiple criteria were used to identify children with suspected ID, reducing the chance of not identifying children at risk for ID. IQ test, one of the criteria used to define ID, is considered the most reliable and valid instrument to measure cognitive abilities, and an essential tool, although not exclusive, to evaluate the presence of ID, respecting cultural values [2].

Children belonging to the group of nonbiological causes have never attended any kind of intervention/stimulation program. Early interventions are designed to support young children who are at risk or who have been identified as having disabilities. In addition, the importance of these interventions, independently of ID etiology, has also been observed [29]. In Brazil, a similar intervention has been successfully documented in a poverty area where mothers were taught to play and stimulate their children [30]. Consequently, it would be expected that a considerable portion of cases could have been potentially reduced if appropriate interventions had been delivered.

## Disclosure Statement

The authors declare that there are no conflicts of interest to disclose.

## Figures and Tables

**Table 1:** ID according to etiologic groups, average IQ score at the age of 4 years by the WPPSI, education, and impairments on adaptive behavior areas^a^

ID group	n (%)	IQ average at 4 years of age (95% CI)	Children at special education	Children with two or more adaptive behavior impairments
Genetic^b^	31 (20.5)	62.5 (60.37–64.55)	52.0%	100.0%
Idiopathic	19 (l2.6)	66.9 (62.07–71.66)	31.5%	100.0%
Neonatal sequelae	20 (13.2)	62.8 (59.61–66.02)	30.0%	100.0%
Other diseases	14 (9.3)	65.6 (62.74–68.38)	21.4%	100.0%
Nonbiological	67 (44.4)	68.4 (65.82–70.92)	0.0%	13.4%

CI = Confidence interval.

a Communication, daily living, and social skills.

b Two individuals were unable to complete the scale.

**Table 2:** ID groups according to demographic and socioeconomic variables, maternal behavior, and health

	Genetic	Idiopathic	Neonatal sequelae	Other diseases	Nonbiological causes	p value
Total number	31	19	20	14	67	0.28
Maternal age						
<19 years	12.9%	42.1%	25.0%	37.5%	24.0%	
20–30 years	45.2%	42.1%	50.0%	43.7%	51.3%	
30–35 years	25.8%	5.3%	10.0%	6.3%	13.3%	
≥36 years	16.1%	10.5%	15.0%	12.5%	11.3%	
Alcohol intake during pregnancy	3.2%	5.3%	5.0%	0.0%	0.0%	0.42
Tobacco smoking during pregnancy	25.8%	31.6%	45.0%	43.8%	40.0%	0.55
Maternal gestational diabetes	3.2%	5.3%	10.0%	6.3%	3.1%	0.76
Maternal depression in the first 4 years	22.6%	42.1%	45.0%	50.0%	24.6%	0.10
Maternal schooling						0.02
0–4 years	17.2%	21.1%	20.0%	56.3%	38.5%	
5–8 years	62.1%	68.4%	60.0%	12.5%	43.1%	
9–11 years	17.2%	10.5%	10.0%	31.3%	16.9%	
≥12 years	3.5%	0.0%	10.0%	0.0%	1.5%	
Paternal schooling						0.20
0–4 years	17.9%	43.8%	17.7%	20.0%	29.3%	
5–8 years	60.7%	43.8%	41.2%	46.7%	50.0%	
9–11 years	14.3%	12.5%	41.2%	20.0%	19.0%	
≥12 years	7.1%	0.0%	0.0%	13.3%	1.7%	
Socioeconomic level						0.07
A	6.7%	0.0%	0.0%	0.0%	0.0%	
B	13.3%	5.6%	0.0%	21.4%	9.7%	
C	50.0%	50.0%	70.0%	42.9%	37.1%	
D	26.7%	44.4%	30.0%	21.4%	41.9%	
E	3.3%	0.0%	0.0%	14.3%	11.3%	
